# Associations of psychotic-like experiences, related symptoms, and working memory with functioning

**DOI:** 10.1192/j.eurpsy.2020.21

**Published:** 2020-02-24

**Authors:** Charlotte A. Chun, Shanna Cooper, Lauren M. Ellman

**Affiliations:** 1 Department of Psychology, Temple University, Philadelphia, Pennsylvania, USA; 2 Department of Mental Health, VA San Diego Healthcare System, San Diego, California, USA; 3 Department of Psychiatry, University of California, San Diego, California, USA

**Keywords:** clinical high risk, cognition, psychosis, role functioning, social functioning, working memory

## Abstract

This study examined the association of spatial working memory and attenuated psychotic-like experiences and related symptoms with social and role functioning. Findings from this study suggest that symptom dimensions and working memory impairment were associated with diminished functioning across a variety of domains. Specifically, negative symptoms and working memory impairment were inversely associated with both social and role functioning, whereas positive and disorganized symptoms showed inverse associations with social functioning only. Symptom dimensions did not moderate cognitive and functional variables, although working memory and attenuated clinical symptoms had an additive effect on functioning. Post-hoc analyses examining symptom dimensions simultaneously showed negative symptoms to be the variable most strongly predictive of overall functioning. These findings suggest that even in a non-clinical sample, sub-threshold psychosis symptoms and cognition may influence people’s social and role functioning.

Social and role functioning play an important role in the continuum of risk for psychosis. People with subclinical schizotypy, clinical high-risk (CHR) for psychosis, and schizophrenia show impairments in premorbid social adjustment, social functioning, and role functioning (in work, school, home, and community roles), which have major implications for quality of life and other domains of functioning [1–6]. Furthermore, poor social and role functioning and stalled social development predict later conversion to psychosis and worse long-term functioning in CHR groups [7–10], suggesting that this is an area of study relevant for clinical practice. However, it is unclear how social functioning is impacted by working memory in subclinical groups on the psychosis spectrum, especially in relation to specific dimensions of subclinical symptoms, including psychotic-like experiences (PLEs or attenuated positive psychotic symptoms) and attenuated negative and disorganized symptoms.

Cognitive deficits correlate with quality of life and functional outcomes, including social functioning, in schizophrenia and CHR samples [11–15]. Further, longitudinal research has indicated that improvement in cognition and symptoms across time predicts better outcomes for social and role functioning in CHR groups [16]. Working memory deficits have been repeatedly found across the psychosis spectrum, including among patients with schizophrenia, CHR samples, and unaffected first-degree relatives of psychosis patients [17–19]. Additionally, meta-analyses suggest that working memory may be one of the few specific cognitive deficits present in subclinical schizotypy groups [20] and that it predicts CHR conversion to psychosis [21,22]. Working memory impairment has been associated with diminished global functioning, social problem-solving skills, and premorbid social adjustment and role functioning in schizophrenia and CHR groups [2,17,23,24]. However, research is lacking on the impact of working memory on social and role functioning in individuals experiencing PLEs and other attenuated symptoms. One study found that working memory deficits were associated with a subscale of a schizotypal personality questionnaire that measures social connections [25], and another study found that objective quality of life showed small associations with overall neurocognitive performance among college students with high schizotypy scores [20]. However, to our knowledge, no study has thoroughly examined social and role functioning specifically in relation to working memory in subclinical psychosis risk.

Positive, negative, and disorganized schizophrenia-spectrum symptoms involve different aspects of cognition, affect, and behavior, and show differential outcomes in these areas on laboratory and experience-sampling measures of factors such as cognitive impairment, emotional experience, and social functioning [26–28]. Meta-analysis studies found that negative—but not positive—clinical symptoms were associated with working memory deficits in patients with schizophrenia [29], whereas positive and negative schizotypy both showed small working memory deficits in subclinical groups [20]. Disorganized symptoms generally show modest associations with working memory, intermediate to those of positive and negative symptoms [30,31]. Overall, however, findings are mixed with some studies finding null or inconsistent associations of working memory across symptom dimensions [32,33].

Negative and disorganized dimensions have been associated with social and role functioning in a variety of studies, and negative symptoms appear to contribute most strongly to functional impairment in schizophrenia and CHR groups, both concurrently and prospectively [24,34–36]. Positive symptoms tend to show the weakest associations with functional outcomes and in individuals at CHR, attenuated positive symptoms may be more strongly related to transition to psychosis than to other correlates of the illness [34]. Subclinical symptom dimensions of psychosis risk are also differentially associated with impairment in different aspects of social functioning. For example, one study found associations between positive schizotypy and peer-relationship problems, and between negative schizotypy and diminished prosocial behavior in a community sample of adolescents [37]. A longitudinal study using latent class analysis showed that a group with consistently high schizotypal traits also had high negative symptoms and poor social functioning over time [38]. Further, there is preliminary evidence that cognitive function may play a role in this relationship: one study found that executive function mediated the relationship between social anhedonia and social impairment in a subclinical group [39]. However, there has been little research on the potential contributing role of working memory toward functioning in subclinical populations.

In sum, there is a large body of research showing that clinical symptoms and neurocognition have major impacts on functioning in schizophrenia-spectrum groups [36,40,41]. However, the complex and possibly interacting relationships among these variables are not well characterized in individuals at putative risk for psychosis who are experiencing PLEs and other attenuated symptoms. Addressing these questions can improve our understanding of what disrupts social and role functioning in psychosis-spectrum psychopathology.

The current study sought to examine how PLEs and attenuated negative and disorganized symptoms and working memory relate to social and role functioning. Additionally, we aimed to investigate whether symptoms and working memory interact to predict functioning. As reviewed above, research has shown that working memory and negative symptoms are associated with one another and both contribute individually to functioning, supporting the hypothesis of an additive model. Further, engagement of working memory capacity requires cognitive effort [42]. Given that motivation and effort are often diminished in people high in negative symptoms, the combination of high negative symptoms and low working memory is expected to be associated with even worse functioning, consistent with the hypothesis of a moderation model.

Hypotheses were made after data collection, but before data analyses, and were pre-registered on Open Science Framework (https://osf.io/4ndwz/). Greater working memory sensitivity and faster reaction times (RT) on the spatial N-back were expected to correlate with better functioning overall and across social and role function subscales. It was hypothesized that negative and disorganized symptoms—but not PLEs—would predict worse N-back performance and lower total and subscale functioning scores. Negative and disorganized symptoms—but not PLEs—were expected to moderate the relationship between N-back performance and overall social and role functioning, such that greater symptoms and worse working memory would be associated with worse functioning. It was expected that these moderation models would fit the data better than additive models of symptom dimensions and working memory predicting social and role function.

## Method

### Participants

Temple University’s Institutional Review Board approved this study. Undergraduate students ages 18^+^ from multiple disciplines at a socioeconomically and racially diverse urban university in the United States could sign up for the study through the university’s online subject pool. After providing informed consent, participants completed questionnaires and cognitive tasks on a laboratory computer in the same testing session. They received course credit for their participation. Assessment of subclinical college samples is advantageous for several reasons: (a) participants’ average age is within the window of typical age of onset of psychosis, (b) subclinical schizotypy predicts later onset of psychotic disorders, (c) it allows for premorbid examination of the correlates of psychotic psychopathology, (d) there are fewer confounds such as medication, stigma, and other effects associated with illness onset, and (e) examination of outcomes in this sample provides a conservative test of our hypotheses given that the sample is generally high functioning [43,44]. Nevertheless, there were substantially more females in the courses that could register for the study and it is possible that findings in our sample will not be generalizable to similarly aged non-college participants, therefore it is necessary for future studies to determine whether these findings can be replicated in community-based samples.

Of participants (*N* = 497) who completed the study, 31 (6%) were excluded due to below-chance performance on the N-back, leaving a final sample of 466 participants. One participant was excluded for low accuracy on the 0-back condition, 10 on the 1-back condition, and 27 on the 2-back condition. Table 1 presents descriptive statistics. As noted in the table, variables had slightly different sample sizes due to missing data on some measures. Gender was significantly associated with all three symptom dimensions, working memory accuracy, and Social Functioning Scale (SFS) social engagement and independence-performance; thus, gender was included as a covariate in correlations among these variables.

**Table 1.** Descriptive characteristics of the final sample

	*N*	Range	Mean (SD)
Age	458	18–36	20.5 (2.8)
Gender	466		
Male			28.3%
Female			71.7%
Race	466		
White			62.2%
Asian/Pacific islander			14.4%
Black or African American			12.2%
More than one race			4.1%
Unknown			7.1%
Ethnicity	466		
Hispanic/Latino			4.3%
Not Hispanic/Latino			95.7%
Maternal education	466		
Less than high school degree			5.4%
High school degree or equivalent			34.1%
Associates degree/two-year college completed			17.5%
Four-year college attended, not completed			4.3%
Bachelor degree			25.8%
Graduate degree			13.1%
Paternal education	466		
Less than high school degree			5.6%
High school degree or equivalent			35.2%
Associates degree/two-year college completed			14.2%
Four-year college attended, not completed			3.0%
Bachelor degree			26.4%
Graduate degree			15.7%
Positive symptoms	466	0–32	6.5 (5.8)
Disorganized symptoms	466	0–6	2.6 (1.8)
Negative symptoms	466	36–108	86.0 (11.2)
0-back RT (correct trials)	466	394.0–951.5	531.5 (96.3)
1-back RT (correct trials)	466	191.4–1194.2	401.6 (158.0)
2-back RT (correct trials)	466	149.7–1088.9	384.2 (153.6)
0-back *d*′	466	−0.06 to 4.91	2.7 (1.0)
1-back *d*′	466	−0.70 to 4.91	3.3 (1.3)
2-back *d*′	466	−0.87 to 4.91	2.3 (1.5)
SFS total	463	91–214	141.2 (19.2)
SFS social engagement	464	3–15	11.0 (2.2)
SFS interpersonal communication	465	3–9	8.3 (1.0)
SFS independence-performance	465	17–39	31.7 (5.1)
SFS recreation	464	5–45	18.5 (6.2)
SFS prosocial	464	3–66	25.3 (10.1)
SFS independence-competence	464	0–39	36.9 (3.2)
SFS occupation	464	9–10	9.5 (.5)

Abbreviations: RT, reaction times; SFS, Social Functioning Scale; SD, standard deviation.

### Materials

#### Prodromal Questionnaire (PQ) [45]

The PQ is a questionnaire designed to identify individuals at risk for psychosis. It has demonstrated concurrent validity with standard interview measures of psychosis risk, and PLEs in particular were found to predict CHR status [45]. Given that factor analytic studies have shown disorganized symptoms to be distinct from PLEs and negative symptoms [46–48], the current study divided PQ positive scale items into separate dimensions of PLEs (PQ items: unusual thinking, paranoia/suspiciousness, and perceptual abnormalities) and disorganized symptoms (PQ items: disorganized thoughts and speech). Items were summed within each dimension to compute continuous scores of symptoms occurring at least once per month. These symptom dimension conceptualizations have been used in previous psychosis risk research [47].

#### Temporal Experience of Pleasure Scale (TEPS) [49]

The TEPS measures self-report of anticipatory and consummatory pleasure and is appropriate for use with schizophrenia-spectrum groups [50]. The TEPS was selected because it taps negative symptoms that are characteristic of psychosis risk without including items directly related to social and role functioning. This allows us to examine associations between negative symptoms and functioning without inflating results from overlapping content. The current study summed items from anticipatory and consummatory subscales to compute continuous scores on a negative symptom dimension (see symptom dimensions in Analyses section below). Because higher scores on the TEPS indicate greater pleasure, scores were reversed for analyses to keep presentation of results consistent across symptom dimensions. Thus, associations described in the results are reported in terms of greater negative symptoms.

#### Social Functioning Scale (SFS) [51]

The SFS is a self-report measure of social and role functioning that is sensitive to functional impairment across the psychosis spectrum [52]. SFS outcomes include a total score and seven subscale scores of social functioning (withdrawal, interpersonal behavior, and prosocial activities) and role functioning (recreation, independence-competence, independence-performance, and occupation). To mitigate issues associated with multiple comparisons, the current study only examined subscale scores in correlations; moderation analyses used the SFS total score.

#### Spatial N-back [53]

The spatial N-back is a working memory task in which participants are instructed to indicate the location of a stimulus displayed in one of four circles fixed in a diamond pattern. There are three conditions: participants indicate the location of the current stimulus in the 0-back condition (control condition), the stimulus one trial back in the 1-back condition (low cognitive load), and the stimulus two trials back in the 2-back condition (high cognitive load). The stimulus duration was 400 ms and the inter-stimulus interval was 1,400 ms. Participants completed a set of practice trials followed by 6 blocks of 7 critical trials per condition, for a total of 126 critical trials. The first trial of every 1-back block and first two trials of every 2-back block were not scored because there were no preceding stimuli to which participants could respond. Average RT was calculated for correct trials only. It was decided a priori that participants who scored below chance (25% accuracy) on any of the three conditions would be excluded. A sensitivity index of performance accuracy was calculated as *d*′ = *Z*_hits_ – *Z*_false alarms_, which is found to be the most appropriate measure of working memory for the N-back in schizophrenia research [54]. Working memory measures for *d*′ and RT were computed by controlling 2-back scores for 0-back scores. For example, 2-back *d*′ was regressed on 0-back d′ and the residuals were saved as a measure of working memory sensitivity.

## Analyses

Symptom dimension conceptualizations and planned analyses were established a priori and pre-registered. Symptom dimensions represent sums of continuous scores within each dimension of PLEs (PQ items: unusual thinking, paranoia/suspiciousness, and perceptual abnormalities), disorganized symptoms (PQ items: disorganized thoughts and speech), and negative symptoms (TEPS anticipatory and consummatory scores). Positive, negative, and disorganized symptoms of psychosis risk exist along a spectrum in the general population and we aimed to examine these symptoms continuously in a subclinical sample to maximize statistical power and avoid the use of artificial cutoffs.

Because all variables except SFS total score deviated from normality (one-sample Kolmogorov–Smirnov test: all other *p* < .001, indicating significant non-normality), associations were examined with Spearman rank-order correlations, which is a non-parametric test appropriate for variables with these type of distributions. To assess for confounding variables, age, gender, race/ethnicity, and parental education were examined in association with symptom dimensions, working memory outcomes, and SFS total and subscale scores. Demographic variables were included as covariates when they were significantly associated with both the independent and dependent variable.

Correlations were conducted among SFS total and subscale scores; PLEs, negative, and disorganized symptom dimensions; and working memory sensitivity and RT. A series of moderation models examined whether PLEs, negative, and disorganized symptoms each respectively moderated the association between working memory sensitivity and overall functioning. These analyses were run independently using hierarchical linear regression and variables were standardized. All residuals from regression models were normally distributed, linear, and heteroscedastic. To reduce the number of analyses, moderation models were run with sensitivity (*d*′) as the only measure of working memory. Additive models were examined when moderation models were not significant. Effect sizes for multiple regressions are reported in terms of *f* ^2^: values below 0.02 represent a negligible effect size and values of 0.02, 0.15, and 0.35^+^ represent small, medium, and large effect sizes, respectively [55].

Given differential findings for anticipatory and consummatory pleasures in the schizophrenia-spectrum literature [49,50], post-hoc analyses examined negative symptom associations separately by TEPS anticipatory and consummatory subscales. Finally, a post-hoc analysis examined PLEs, negative, and disorganized symptoms as independent variables simultaneously predicting SFS total scores to examine which symptom dimensions were most strongly predictive of functioning.

## Results

Table 2 presents Spearman rank-order correlations. PLEs and disorganized symptoms showed small associations with diminished functioning for social engagement, interpersonal communication, and independence-competence. Negative symptoms showed small associations with diminished social engagement, recreation, prosocial, and overall functioning. None of the symptom dimensions were associated with working memory *d*′ or RT. Working memory sensitivity showed small associations with diminished recreation, independence-performance, and overall social and role functioning. Working memory RT was not associated with any measures of functioning on the SFS.

**Table 2.** Spearman’s rank-order correlations between N-back variables, subclinical symptoms, and SFS outcomes (*N* = 466)

	Soc Engag^a^	Interp Comm^b^	Indep-Perf^b^	Recr^a^	Prosoc^a^	Indep-Comp^a^	Occup^a^	PLEs	Dis Sx	Neg Sx	WM *d*′	WM RT
SFS Total^c^	0.38**	0.31**	0.67**	0.74**	0.84**	0.41**	0.20**	−0.06	−0.04	−0.19**	−0.11*	0.05
Soc Engag^a^		0.42**	0.14**	0.14**	0.29**	0.18**	0.15**	−0.12*	−0.12*	−0.13*	−0.04	0.008
Interp-Comm^b^			0.20**	0.03	0.22**	0.25**	0.08	−0.13**	−0.17**	−0.10*	0.02	0.03
Indep-Perf^b^				0.42**	0.32**	0.44**	0.19**	−0.07^d^	−0.08^d^	−0.18** ^d^	−0.11* ^d^	0.007
Recr^a^					0.51**	0.19**	0.07	0.03	0.04	−0.16**	−0.10*	0.04
Prosoc^a^						0.14**	0.14**	−0.04	−0.01	−0.12*	−0.08	0.04
Indep-Comp^a^							0.24**	−0.13**	−0.14**	−0.07	−0.01	0.002
Occup^a^								0.001	−0.06	0.07	0.02	−0.003
PLEs									0.71**	0.11*	−0.03	0.004
Dis Sx										0.12**	−0.03	−0.02
Neg Sx											−0.06	0.02
WM *d*′												−0.26**

Abbreviations: Dis, disorganized; Indep-Comp, independence-competence; Indep-Perf, independence-performance; Interp Comm, interpersonal communication; Neg, negative; Occup, occupation; PLEs, psychotic-like experiences; Prosoc, prosocial; Recr, recreation; SFS, Social Functioning Scale; Soc Engag, social engagement; Sx, symptoms; WM *d*′ = working memory sensitivity. WM RT, working memory reaction time.

a *N* = 464.

b *N* = 465.

c *N* = 463.

d Gender as covariate.

* *p* < .05

** *p* < .01.

Table 3 presents moderation models. Three separate hierarchical linear regression models examined were conducted for each of the three symptom clusters as independent variables predicting overall functioning on the SFS, in conjunction with working memory (additive model), and in interaction with working memory (moderation model). Moderation models are displayed in Step 2. Contrary to hypotheses, none of the symptom dimensions moderated the relationship between working memory sensitivity and overall social and role functioning.

**Table 3.** Hierarchical linear regression models of symptom dimensions and working memory as independent variables predicting total social and role functioning (*N* = 463)

Step 1 (*df* = 2, 460)									Step 2 (*df* = 1, 459)				
Symptom dimension				WM *d*′			Step 1 total (additive model)		Symptom dimension × WM d′ (moderation model)				
	*B*_1_	95% CI (*B*_1_)	*β*_1_	*B*_2_	95% CI (*B*_2_)	*β*_2_	*f^2^*	*p*	*B*	95% CI (*B*)	*β*	*f^2^*	*p*
(1) PLEs	−0.13	(−1.88, 1.62)	−0.007	−2.37	(−4.11, −0.62)	−0.12	0.015	.03	0.05	(−1.71, 1.81)	0.003	0.000	.95
(2) Dis	−1.09	(−2.84, 0.65)	−0.06	−2.38	(−4.12, −0.64)	−0.12	0.019	.01	−1.09	(−2.83, 0.66)	0.01	0.000	.87
(3) Neg	4.79	(3.09, 6.49)	−0.25	−2.67	(−4.36, −0.98)	−0.14	0.083	<.0001	0.23	(−1.43, 1.88)	0.09	0.000	.79

Values of *f* ^2^ and *p* for Step 2 reflect effect size and significance for the change in variance explained for Step 2 over and above Step 1. Three separate hierarchical regressions were conducted: (1) the additive model of PLEs and working memory as independent variables predicting SFS Total scores at Step 1, and the interaction of PLEs and working memory predicting SFS Total scores at Step 2 (testing whether the interaction term explains additional variance over and above the additive model); (2) the additive model of disorganized symptoms and working memory as independent variables predicting SFS Total scores at Step 1 and the interaction of disorganized symptoms and working memory predicting SFS total scores at Step 2; and (3) the additive model of negative symptoms and working memory as independent variables predicting SFS total scores at Step 1 and the interaction of negative symptoms and working memory predicting SFS total scores at Step 2.

Abbreviations: *B*, unstandardized coefficient. *β*, standardized coefficient; CI, confidence intervals; Dis, disorganized symptoms; Neg, negative symptoms; PLEs, psychotic-like experiences; WM, working memory.

The additive models were all significant, but effects were subtle: PLEs and working memory (negligible effect size), disorganized symptoms and working memory (negligible effect size), and negative symptoms and working memory (small effect size) all predicted SFS total scores. The simultaneous regressions of working memory and symptom dimensions and their additive effects are displayed in the table at Step 1. The additive effects of working memory and each of the three symptom dimensions significantly predicted social functioning. With working memory and PLEs in the model simultaneously, working memory was the only independent variable that significantly predicted functioning. Similarly, with working memory and disorganized symptoms both in the model, only working memory was significant. This indicates that these symptoms do not add significant predictive value in functioning over-and-above working memory. In contrast, both working memory and negative symptoms significantly predicted social functioning when included simultaneously.

### Post-hoc analyses

Post-hoc analyses examining associations with TEPS anticipatory and consummatory subscales independently showed similar results to the combined negative symptom dimension (see Table 4). Anticipatory anhedonia showed small associations with poor social engagement, interpersonal communication, recreation, prosocial, and overall social and role functioning. Consummatory anhedonia showed small associations with poor social engagement, recreation, and overall functioning. Neither TEPS subscale correlated with working memory sensitivity or RT.

**Table 4.** Post-hoc analyses: Spearman’s rank-order correlations using TEPS subscales (*N* = 466)

	Anticipatory anhedonia	Consummatory anhedonia
SFS social engagement^a^	−0.14**	−0.10*
SFS interpersonal communication^e^	−0.13**	−0.06
SFS independence-performance^b^ ^,^^c^	−0.18**	−0.15**
SFS recreation^a^	−0.12**	−0.16**
SFS prosocial^a^	−0.15**	−0.07
SFS independence-competence^a^	−0.08	−0.06
SFS occupation^a^	−0.07	−0.05
SFS total score^d^	−0.20**	−0.16**
WM *d*′	−0.03	−0.08
WM reaction time	0.001	0.03

Higher TEPS anticipatory and consummatory anhedonia scores indicate more severe negative symptoms. Higher SFS scores indicate better functioning. Higher WM *d*′ and lower (quicker) WM reaction times indicate better working memory performance.

Abbreviations: SFS, Social Functioning Scale; WM, working memory.

a *N* = 464.

b *N* = 462.

c Gender included as covariate.

d *N* = 463.

e *N* = 465.

* *p* < .05.

** *p* < .01.

Post-hoc analyses examined PLEs, negative, and disorganized symptoms as independent variables simultaneously predicting SFS total score using a single linear regression model (see Table 5). With all symptom dimensions in the model concurrently, negative symptoms was the only independent variable significantly predicting overall functioning, at the level of a small effect size.

**Table 5.** Post-hoc analyses: linear regression of symptom dimensions as independent variables simultaneously predicting social and role functioning (*N* = 463)

	*B*	95% CI (B)	SE (*B*)	*β*	*f* ^2^	Total *R* ^2^	Total *F*
PLEs	0.23	(−0.17, 0.64)	0.21	0.07	0.003	0.06	9.95*
Disorganized symptoms	−0.82	(−2.13, 0.49)	0.67	−0.08	0.003		
Negative symptoms	−0.41	(0.25, 0.56)	0.08	−0.24*	0.06		

A regression was conducted of the three symptom clusters as independent variables jointly predicting SFS total scores.

Abbreviations: CI, confidence intervals; PLEs, psychotic-like experiences; SE, standard error.

* *p* < .01.

## Discussion

Findings from this study suggest that PLEs, negative and disorganized symptoms and poor working memory sensitivity are associated with diminished functioning across a variety of domains. PLEs and disorganized symptoms were inversely associated with social functioning and negative symptoms were inversely associated with both social and role functioning, consistent with previous findings from studies assessing subclinical schizotypy [37,56]. These results are also consistent with findings that poor social and role functioning are associated with CHR status [10] and conversion to psychosis [9].

Symptom dimensions did not moderate cognitive and functional variables, although working memory and attenuated clinical symptoms had additive effects on functioning. Post-hoc analyses suggested that of the attenuated clinical symptom dimensions, negative symptoms was the independent variable most strongly predictive of overall functioning, consistent with previous research [34,36,57]. Additionally, negative symptoms and working memory each contributed unique variance to social and role functioning, consistent with previous research in a CHR sample [36]. Overall, results from this study suggest that even at an attenuated level, symptoms and cognition are significantly associated with functioning in social interactions and work or community roles.

Associations between symptoms and functioning were small, especially compared to a meta-analysis that found very large effect sizes for functional differences between CHR and healthy control groups [58]. The subtle effects in our study could be attributed to the use of an undergraduate sample, which is expected to have high levels of social, role, and cognitive functioning on average. Further, university life provides structure and accessibility for engagement in social, recreational, and community roles. The small effects in our study may also be partially attributed to our examination of symptoms dimensionally, instead of restricting analyses to clinically relevant symptoms. Nevertheless, average SFS subscale scores from our sample were comparable to those from a previous study assessing social and role functioning in college undergraduates across a range of subclinical schizotypy scores [59].

The current study was correlational; thus, the mechanisms through which negative symptoms and working memory affect social and role functioning are still not well understood. A parsimonious explanation is that there is often overlap in content between measurement of negative symptoms and cognitive, social, and role functioning [60,61]. For example, the Scale for the Assessment of Negative Symptoms (SANS) [62] includes items assessing impaired attention, and the Clinical Assessment Interview for Negative Symptoms (CAINS) [63] includes items assessing motivation and pleasure for social, recreational, and vocational activities. However, some of these concerns are likely mitigated in the current study, given that the TEPS focuses largely on physical anhedonia, which has little overlap with SFS content or cognitive abilities.

An alternative hypothesis is that negative symptoms and working memory impairment may make social, occupational, and recreational activities more difficult, leading to withdrawal when tasks or interactions are perceived as too effortful and not sufficiently rewarding [44,64–66]. Instrumental activities of daily living such as managing food preparation, household chores, and finances are complex tasks that involve keeping goals in mind and remembering details while planning and executing multi-step processes [67]. Not surprisingly, these tasks are associated with executive functions such as working memory [67,68], which require cognitive effort [42]. Social interactions similarly involve a complex set of processes. For example, conversations may involve identifying other people’s goals, interests, and beliefs; keeping track of what has been said; and integrating this information while generating ideas to discuss, following social conventions, and inhibiting inappropriate behavior [69,70]. Engagement in social activities taps executive functioning, emotional, and motivational resources [70–72]. Thus, diminished motivation and working memory impairment may jointly make it difficult for people at risk for psychosis to be successful in social and role functioning activities.

The current study primarily investigated how cognitive variables relate to functioning. We did not aim to measure motivational variables or affective variables such as the experience and expression of emotions like happiness, sadness, or fear; however, the addition of such variables would be important for future studies to include. Previous findings indicate that affective variables likely play an important role in social functioning (see [69] for review). Further, affective factors may interact with cognitive factors. For example, one study showed that working memory moderated the relationship between self-report of physical anhedonia and intensity of emotional experience to positive stimuli in patients with schizophrenia and non-psychiatric controls. That is, people with better working memory showed stronger negative associations between anhedonia and emotional response to pleasant events [73]. Patients with schizophrenia may engage in fewer goal-directed and pleasurable activities due to decreased motivation associated with abnormalities in reward processing, and accompanying beliefs that they will not enjoy these activities [74,75]. Similar processes may influence the relationship between attenuated negative symptoms and functioning in subclinical groups [76,77]; however, overlapping mechanisms were not investigated in the current study and should be examined in future investigations. In summary, cognitive and affective variables likely both contribute to social and role functioning and may interact to predict functioning. Future studies may benefit from assessing cognition, affect, and functioning simultaneously across a range of impairment in psychosis-spectrum psychopathology.

Unexpectedly, symptom dimensions were not associated with working memory performance in the current study. This contrasts findings from studies in patients with schizophrenia [29], but is consistent with other schizophrenia-spectrum research [33]. Null findings could be attributed to the symptom measures used in the current study; for example, disorganized symptoms were assessed through self-report, whereas many other studies have used semi-structured interviews (e.g., the Structured Interview for Prodromal Symptoms; [78]) or behavior-based measures of natural speech (e.g., the Communication Disturbances Index [79]).

The current study had a number of limitations. The SFS primarily assesses retrospective report of the frequency of social and role activities. Future research on the relationship between cognition and functioning would benefit from using ecological momentary assessment to capture quantity and quality of social, role, and cognitive functioning in the moment [80]. The SFS was originally developed to assess social and role functioning in people with schizophrenia; thus, some of the subscales are not particularly relevant for many college students (e.g., employment/occupation). There are few questionnaires assessing social and role functioning designed with subclinical samples in mind but interview-based measures such as the Global Functioning: Social and Global Functioning: Role interviews [81] would be useful in future studies. Due to the high-functioning nature of the undergraduate sample assessed, SFS scores generally approached ceiling—with the exception of recreation and prosocial subscales—which may have limited our ability to find associations. Nonetheless, this strengthens the impact of the findings that symptoms and cognition were associated with functioning in this college sample. The current study used self-report measures of subclinical symptoms and findings from a meta-analysis suggest that these types of self-reports may be influenced by overestimation [82]. Because participants signed up for the study of their own accord and were not systematically recruited, self-selection biases cannot be ruled out. Our sample was drawn from a public university with a relatively diverse body of students in terms of socioeconomic and ethnic/racial composition; however, effects may be even stronger in a community sample. Finally, there was a higher proportion of females to males in our study, although results held after controlling for gender.

## Conclusions

Social functioning, cognitive deficits, and negative symptoms are more stable and traditionally more treatment-resistant than other symptoms and sequelae of CHR and schizophrenia [9,83–86]. The current study found that working memory and subclinical symptoms, especially negative symptoms, are associated with social and role functioning in individuals at putative risk for psychosis. Because social functioning and impairment are key variables predicting conversion to psychosis [7], early identification is crucial. Opportunities for psychosocial and cognitive intervention may mitigate functional decline and improve long-term outcomes.

## Financial support

Funding for this study was provided by Temple University’s College of Liberal Arts Research Award, a University Start-Up Fund awarded to Lauren M. Ellman, PhD, and the following grant awarded to Lauren M. Ellman from NIMH: R01MH112613.

## References

[1] Addington J, Penn D, Woods S, Addington D, Perkins D. Social functioning in individuals at clinical high risk for psychosis. Schizophr Res. 2007;99(1–3):119–124. 18023329 10.1016/j.schres.2007.10.001 PMC2292799

[2] Bucci P, Galderisi S, Mucci A, Rossi A, Rocca P, Bertolino A, et al. Premorbid academic and social functioning in patients with schizophrenia and its associations with negative symptoms and cognition. Acta Psychiatr Scand. 2018;138(3):253–266. 29984409 10.1111/acps.12938

[3] McCleery A, Divilbiss M, St-Hilaire A, Aakre J, Seghers J, Bell E, et al. Predicting social functioning in schizotypy: an investigation of the relative contributions of theory of mind and mood. J Nerv Ment Dis. 2012;200(2):47–152.doi: 10.1371/journal.pone.0134936. PMC4431998 22297312

[4] Pelletier-Baldelli A, Bernard J, Mittal V. Intrinsic functional connectivity in salience and default mode networks and aberrant social processes in youth at ultra-high risk for psychosis. Plos One. 2015;10(8):e0134936. 26252525 10.1371/journal.pone.0134936 PMC4529226

[5] Takahashi T, Higuchi Y, Komori Y, Nishiyama S, Nakamura M, Sasabayashi D, et al. Quality of life in individuals with attenuated psychotic symptoms: Possible role of anxiety, depressive symptoms, and socio-cognitive impairments. Psychiatry Res. 2017;257:431–437. 28837932 10.1016/j.psychres.2017.08.024

[6] Thermenos H, Juelich R, DiChiara S, Mesholam-Gately R, Woodberry K, Wojcik J, et al. Hyperactivity of caudate, parahippocampal, and prefrontal regions during working memory in never-medicated persons at clinical high-risk for psychosis. Schizophr Res. 2016;173(1–2):1–12. 26965745 10.1016/j.schres.2016.02.023 PMC4836956

[7] Cannon T, Cadenhead K, Cornblatt B, Woods S, Addington J, Walker E, et al. Prediction of psychosis in youth at high clinical risk: a multisite longitudinal study in North America. Arch Gen Psychiatry. 2008;65(1):28–37. 18180426 10.1001/archgenpsychiatry.2007.3 PMC3065347

[8] Carrión R, McLaughlin D, Goldberg T, Auther A, Olsen R, Olvet D, et al. Prediction of functional outcome in individuals at clinical high risk for psychosis. JAMA Psychiatry. 2013;70(11):1133–1142. 24006090 10.1001/jamapsychiatry.2013.1909 PMC4469070

[9] Cornblatt B, Carrión R, Addington J, Seidman L, Walker E, Cannon T, et al. Risk factors for psychosis: impaired social and role functioning. Schizophr Bull. 2012;38(6):1247–1257. 22080497 10.1093/schbul/sbr136 PMC3494064

[10] Velthorst E, Zinberg J, Addington J, Cadenhead K, Cannon T, Carrión R, et al. Potentially important periods of change in the development of social and role functioning in youth at clinical high risk for psychosis. Dev Psychopathol. 2018;30(1):39–47. 28420458 10.1017/S0954579417000451 PMC5648633

[11] Halverson T, Orleans-Pobee M, Merritt C, Sheeran P, Fett A-K, Penn D. Pathways to functional outcomes in schizophrenia spectrum disorders: meta-analysis of social cognitive and neurocognitive predictors. Neurosci Biobehav Rev. 2019;105:212–219. 31415864 10.1016/j.neubiorev.2019.07.020

[12] Bechi M, Bosia M, Spangaro M, Buonocore M, Cavedoni S, Agostoni G, et al. Exploring functioning in schizophrenia: predictors of functional capacity and real-world behaviour. Psychiatry Res. 2017;251:118–124. 28199909 10.1016/j.psychres.2017.02.019

[13] Lin A, Wood S, Nelson B, Brewer W, Spiliotacopoulos D, Bruxner A, et al. Neurocognitive predictors of functional outcome two to 13 years after identification as ultra-high risk for psychosis. Schizophr Res. 2011;132(1):1–7. 21763109 10.1016/j.schres.2011.06.014

[14] Niendam T, Bearden C, Johnson J, McKinley M, Loewy R, O'Brien M, et al. Neurocognitive performance and functional disability in the psychosis prodrome. Schizophr Res. 2006;84(1):100–111. 16563699 10.1016/j.schres.2006.02.005

[15] Tolman A, Kurtz M. Neurocognitive predictors of objective and subjective quality of life in individuals with schizophrenia: a meta-analytic investigation. Schizophr Bull. 2012;38(2):304–315. 20624752 10.1093/schbul/sbq077 PMC3283161

[16] Niendam T, Bearden C, Zinberg J, Johnson J, O'Brien M, Cannon T. The course of neurocognition and social functioning in individuals at ultra high risk for psychosis. Schizophr Bull. 2007;33(3):772–781. 17420177 10.1093/schbul/sbm020 PMC2526130

[17] Goghari V, Brett C, Tabraham P, Johns L, Valmaggia L, Broome M, et al. Spatial working memory ability in individuals at ultra high risk for psychosis. J Psychiatr Res. 2014;50:100–105. 24398256 10.1016/j.jpsychires.2013.12.010 PMC4127476

[18] Seidman L, Shapiro D, Stone W, Woodberry K, Ronzio A, Cornblatt B, et al. Association of neurocognition with transition to psychosis baseline functioning in the second phase of the North American prodrome longitudinal study. JAMA Psychiatry. 2016;73(12):1239–1248. 27806157 10.1001/jamapsychiatry.2016.2479 PMC5511703

[19] Snitz B, MacDonald A, Carter C. Cognitive deficits in unaffected first-degree relatives of schizophrenia patients: a meta-analytic review of putative endophenotypes. Schizophr Bull. 2006;32(1):179–194. 16166612 10.1093/schbul/sbi048 PMC2632195

[20] Chun C, Minor K, Cohen A. Neurocognition in psychometrically defined college schizotypy samples: we are NOT measuring the "right stuff". J Int Neuropsychol Soc. 2013;19(3):324–337. 23448879 10.1017/S135561771200152X

[21] De Herdt A, Wampers M, Vancampfort D, De Hert M, Vanhees L, Demunter H, et al. Neurocognition in clinical high risk young adults who did or did not convert to a first schizophrenic psychosis: a meta-analysis. Schizophr Res. 2013;149(1–3):48–55. 23830855 10.1016/j.schres.2013.06.017

[22] Fusar-Poli P, Deste G, Smieskova R, Barlati S, Yung A, Howes O, et al. Cognitive functioning in prodromal psychosis: a meta-analysis. Arch Gen Psychiatry. 2012;69(6):562–571. 22664547 10.1001/archgenpsychiatry.2011.1592

[23] Huang J, Tan S, Walsh S, Spriggens L, Neumann D, Shum D, et al. Working memory dysfunctions predict social problem solving skills in schizophrenia. Psychiatry Res. 2014;220(1–2):96–101. 25110314 10.1016/j.psychres.2014.07.043

[24] Vesterager L, Christensen T, Olsen B, Krarup G, Melau M, Forchhammer H, et al. Cognitive and clinical predictors of functional capacity in patients with first episode schizophrenia. Schizophr Res. 2012;141(2–3):251–256. 23017825 10.1016/j.schres.2012.08.023

[25] Park S, McTigue K. Working memory and the syndromes of schizotypal personality. Schizophr Res. 1997;26(2):213–220. 9323353 10.1016/s0920-9964(97)00051-0

[26] Barrantes-Vidal N, Chun C, Myin-Germeys I, Kwapil T. Psychometric schizotypy predicts psychotic-like, paranoid, and negative symptoms in daily life. J Abnorm Psychol. 2013;122(4):1077–1087. 24364610 10.1037/a0034793

[27] Kwapil T, Barrantes-Vidal N. Schizotypal personality disorder: an integrative review In: Widiger T, editor. The Oxford handbook of personality disorders, Oxford: Oxford University Press; 2012; p. 437–477.

[28] Gross G, Kwapil T, Burgin C, Raulin M, Silvia P, Kemp K, et al. Validation of the multidimensional schizotypy scale-brief in two large samples. J Psychopathol Behav Assess. 2018;40(4):669–677.

[29] Ventura J, Hellemann G, Thames A, Koellner V, Nuechterlein K. Symptoms as mediators of the relationship between neurocognition and functional outcome in schizophrenia: a meta-analysis. Schizophr Res. 2009;113(2–3):189–199. 19628375 10.1016/j.schres.2009.03.035 PMC2825750

[30] Lindsberg J, Poutiainen E, Kalska H. Clarifying the diversity of first-episode psychosis: neuropsychological correlates of clinical symptoms. Nordic J Psychiatry. 2009;63(6):493–500. 10.3109/08039480903118182 19685368

[31] Ventura J, Thames A, Wood R, Guzik L, Hellemann G. Disorganization and reality distortion in schizophrenia: a meta-analysis of the relationship between positive symptoms and neurocognitive deficits. Schizophr Res. 2010;121(1–3):1–14. 20579855 10.1016/j.schres.2010.05.033 PMC3160271

[32] Forbes N, Carrick L, McIntosh A, Lawrie S. Working memory in schizophrenia: a meta-analysis. Psychol Med. 2009;39(6):889–905. 18945379 10.1017/S0033291708004558

[33] Yong E, Barbato M, Penn D, Keefe R, Woods S, Perkins D, et al. Exploratory analysis of social cognition and neurocognition in individuals at clinical high risk for psychosis. Psychiatry Res. 2014;218(1–2):39–43. 24755041 10.1016/j.psychres.2014.04.003 PMC4062969

[34] Carrión R, Demmin D, Auther A, McLaughlin D, Olsen R, Lencz T, et al. Duration of attenuated positive and negative symptoms in individuals at clinical high risk: associations with risk of conversion to psychosis and functional outcome. J Psychiatr Res. 2016;81:95–101. 27424062 10.1016/j.jpsychires.2016.06.021 PMC5021595

[35] Fulford D, Niendam T, Floyd E, Carter C, Mathalon D, Vinogradov S, et al. Symptom dimensions and functional impairment in early psychosis: more to the story than just negative symptoms. Schizophr Res. 2013;147(1):125–131. 23587696 10.1016/j.schres.2013.03.024 PMC3663589

[36] Meyer E, Carrión R, Cornblatt B, Addington J, Cadenhead K, Cannon T, NAPLS Group The relationship of neurocognition and negative symptoms to social and role functioning over time in individuals at clinical high risk in the first phase of the North American Prodrome Longitudinal Study. Schizophr Bull. 2014;40(6):1452–1461. 24550526 10.1093/schbul/sbt235 PMC4193704

[37] Abu-Akel A, Baxendale L, Mohr C, Sullivan S. The association between schizotypal traits and social functioning in adolescents from the general population. Psychiatry Res. 2018;270:895–900. 30551341 10.1016/j.psychres.2018.11.007

[38] Wang Y, Shi H, Liu W, Xie D, Geng F, Yan C, et al. Trajectories of schizotypy and their emotional and social functioning: an 18-month follow-up study. Schizophr Res. 2018;193:384–390. 28751128 10.1016/j.schres.2017.07.038

[39] Tully L, Lincoln S, Hooker C. Attentional control mediates the relationship between social anhedonia and social impairment. Frontiers Psychol. 2014;5(1):1384. 10.3389/fpsyg.2014.01384 PMC4255878 25538647

[40] Foussias G, Mann S, Zakzanis K, Van Reekum R, Agid O, Remington G. Prediction of longitudinal functional outcomes in schizophrenia: the impact of baseline motivational deficits. Schizophr Res. 2011;132(1):24–27. 21771567 10.1016/j.schres.2011.06.026

[41] Green M, Kern R, Heaton R. Longitudinal studies of cognition and functional outcome in schizophrenia: implications for MATRICS. Schizophr Res. 2004;72(1):41–51. 15531406 10.1016/j.schres.2004.09.009

[42] Gerritsen C, Maheandiran M, Lepock J, Ahmed S, Kiang M, Bagby R, et al. Negative symptoms in the clinical high-risk state for psychosis: connection with cognition and primacy in impacting functioning. Early Interv Psychiatry. 2019. doi: 10.1111/eip.12843 [Epub ahead of print]. 31264790

[43] Kwapil T, Barrantes-Vidal N. Schizotypy: looking back and moving forward. Schizophr Bull. 2015;41(S2):S366–S373. 25548387 10.1093/schbul/sbu186 PMC4373633

[44] Luther L, Salyers M, Firmin R, Marggraf M, Davis B, Minor K. Additional support for the cognitive model of schizophrenia: evidence of elevated defeatist beliefs in schizotypy. Compr Psychiatry. 2016;68:40–47. 27234181 10.1016/j.comppsych.2016.03.006

[45] Loewy R, Bearden C, Johnson J, Raine A, Cannon T. The prodromal questionnaire (PQ): preliminary validation of a self-report screening measure for prodromal and psychotic syndromes. Schizophr Res. 2005;79(1):117–125. 16276559

[46] Arndt S, Alliger R, Andreasen N. The distinction of positive and negative symptoms: the failure of a two-dimensional model. Br J Psychiatry. 1991;158(3):317–322. 2036528 10.1192/bjp.158.3.317

[47] Cooper S, Klugman J, Heimberg R, Anglin D, Ellman L. Attenuated positive psychotic symptoms and social anxiety: along a psychotic continuum or different constructs? Psychiatry Res. 2016;235:139–147. 26657307 10.1016/j.psychres.2015.11.027

[48] Raine A, Reynolds C, Lencz T, Scerbo A, Triphon N, Kim D. Cognitive-perceptual, interpersonal and disorganized features of schizotypal personality. Schizophr Bull. 1994;20(1):191–201. 8197415 10.1093/schbul/20.1.191

[49] Gard D, Gard M, Kring A, John O. Anticipatory and consummatory components of the experience of pleasure: a scale development study. J Res Personality. 2006;40(6):1086–1102.

[50] Cooper S, Kring A, Ellman L. Attenuated positive psychotic symptoms and the experience of anhedonia. Early Interv Psychiatry. 2018;12(6):1188–1192. 28597553 10.1111/eip.12439 PMC5723249

[51] Birchwood M, Smith J, Cochrane R, Wetton S, Copestake S. The social functioning scale: the development and validation of a new scale of social adjustment for use in family intervention programmes with schizophrenic patients. Br J Psychiatry. 1990;157(6):853–859. 2289094 10.1192/bjp.157.6.853

[52] Hajdúk M, Klein H, Harvey P, Penn D, Pinkham A. Paranoia and interpersonal functioning across the continuum from healthy to pathological: network analysis. Br J Clin Psychol. 2019;58(1):19–34. 30028025 10.1111/bjc.12199 PMC6339834

[53] Callicott J, Ramsey N, Tallent K, Bertolino A, Knable M, Cappola R, et al. Functional magnetic resonance imaging brain mapping in psychiatry: methodological issues illustrated in a study of working memory in schizophrenia. Neuropsychopharmacology. 1998;18(3):186–196. 9471116 10.1016/S0893-133X(97)00096-1

[54] Haatveit B, Sundet K, Hugdahl K, Ueland T, Melle I, Andreassen O. The validity of d prime as a working memory index: results from the “Bergen n-back task”. J Clin Exp Neuropsychol. 2010;32(8):871–880. 20383801 10.1080/13803391003596421

[55] Cohen J. Statistical power analysis for the behavioral sciences, 2nd ed. Hillsdale, NJ: L. Erlbaum Associates; 1988.

[56] Fonseca-Pedrero E, Lemos-Giráldez S, Paíno-Piñeiro M, Villazón-García U, Muñiz J. Schizotypal traits, obsessive-compulsive symptoms, and social functioning in adolescents. Compr Psychiatry. 2010;51(1):71–77. 19932829 10.1016/j.comppsych.2009.02.003

[57] Schlosser D, Campellone T, Biagianti B, Delucchi K, Gard D, Fulford D, et al. Modeling the role of negative symptoms in determining social functioning in individuals at clinical high risk of psychosis. Schizophr Res. 2015;169(1–3):204–208. 26530628 10.1016/j.schres.2015.10.036 PMC4681660

[58] Fusar-Poli P, Rocchetti M, Sardella A, Avila A, Brandizzi M, Caverzasi E, et al. Disorder, not just state of risk: meta-analysis of functioning and quality of life in people at high risk of psychosis. Br J Psychiatry. 2015;207(3):198–206. 26329563 10.1192/bjp.bp.114.157115

[59] Dinzeo T, Serna V, Pujji S, Sledjeski E. The relationship of categorical and phonological verbal fluency to negative schizotypy and social functioning in a non-clinical sample. Cogn Neuropsychiatry. 2018;23(1):43–57. 29258396 10.1080/13546805.2017.1418307

[60] Lyne J, O'Donoghue B, Roche E, Renwick L, Cannon M, Clarke M. Negative symptoms of psychosis: a life course approach and implications for prevention and treatment. Early Interv Psychiatry. 2018;12(4):561–571. 29076240 10.1111/eip.12501

[61] Lincoln T. Current developments and challenges in the assessment of negative symptoms. Schizophr Res. 2017;186:8–18. 26960948 10.1016/j.schres.2016.02.035

[62] Andreasen N. Negative symptoms in schizophrenia: definition and validation. Arch Gen Psychiatry. 1982;39(1):784–788. 7165477 10.1001/archpsyc.1982.04290070020005

[63] Kring A, Gur R, Blanchard J, Horan W, Reise S. The Clinical Assessment Interview for Negative Symptoms (CAINS): final development and validation. Am J Psychiatry. 2013;170(2):165–172. 23377637 10.1176/appi.ajp.2012.12010109 PMC3785242

[64] Degnan A, Berry K, Sweet D, Abel K, Crossley N, Edge D. Social networks and symptomatic and functional outcomes in schizophrenia: a systematic review and meta-analysis. Soc Psychiatry Psychiatr Epidemiol. 2018;53(9):873–888.doi: 10.1007/s00127-018-1552-8. 29951929 PMC6133157

[65] DeRosse P, Barber A, Fales C, Malhotra A. Deconstructing avolition: initiation vs. persistence of reward-directed effort. Psychiatry Res. 2019;273:647–652. 31207847 10.1016/j.psychres.2019.01.073 PMC7864548

[66] Thorup A, Petersen L, Jeppesen P, Øhlenschlæger J, Christensen T, Krarup G, et al. Social network among young adults with first-episode schizophrenia spectrum disorders. Soc Psychiatry Psychiatr Epidemiol. 2006;41(10):761–770. 16900304 10.1007/s00127-006-0098-3

[67] Borella E, Cantarella A, Joly E, Ghisletta P, Carbone E, Coraluppi D, et al. Performance-based everyday functional competence measures across the adult lifespan: the role of cognitive abilities. Int Psychogeriatr. 2017;29(12):2059–2069. 28595679 10.1017/S1041610217000680

[68] Burton C, Strauss E, Hultscha D, Huntera M. Cognitive functioning and everyday problem solving in older adults. Clin Neuropsychol. 2006;3:432–452. 10.1080/13854040590967063 16895857

[69] Porcelli S, Van der Wee N, Van der Werff S, Aghajani M, Glennon J, Van Heukelum S, et al. Social brain, social dysfunction and social withdrawal. Neurosci Biobehav Rev. 2019;97:10–33. 30244163 10.1016/j.neubiorev.2018.09.012

[70] Ybarra O, Winkielman P. On-line social interactions and executive functions. Frontiers Hum Neurosci. 2012;6:75. 10.3389/fnhum.2012.00075 PMC3321651 22509160

[71] Dodell-Feder D, Germine L. Epidemiological dimensions of social anhedonia. Clin Psychol Sci. 2018;6(5):735–743.

[72] Hari R, Henriksson L, Malinen S, Parkkonen L. Centrality of social interaction in human brain function. Neuron. 2015;88(1):181–193. 26447580 10.1016/j.neuron.2015.09.022

[73] Burbridge J, Barch D. Anhedonia and the experience of emotion in individuals with schizophrenia. J Abnorm Psychol. 2007;116(1):30–42. 17324014 10.1037/0021-843X.116.1.30

[74] Gold J, Waltz J, Prentice K, Morris S, Heerey E. Reward processing in schizophrenia: a deficit in the representation of value. Schizophr Bull. 2008;34(5):835–847. 18591195 10.1093/schbul/sbn068 PMC2518641

[75] Strauss G, Gold J. A new perspective on anhedonia in schizophrenia. Am J Psychiatry. 2012;169(4):364–373. 22407079 10.1176/appi.ajp.2011.11030447 PMC3732829

[76] Fervaha G, Zakzanis K, Jeffay E, Graff-Guerrero A, Foussias G, Agid O, et al. Amotivation as central to negative schizotypy and their predictive value for happiness. Pers Individ Differ. 2014;68:37–42.

[77] Li X, Xiao Y, Zou L, Li H, Yang Z, Shi H, et al. The effects of working memory training on enhancing hedonic processing to affective rewards in individuals with high social anhedonia. Psychiatry Res. 2016;245:482–490. 27639163 10.1016/j.psychres.2016.09.006

[78] McGlashan T, Walsh B, Woods S. The psychosis-risk syndrome handbook for diagnosis and follow-up. New York: Oxford University Press; 2010.

[79] Docherty N. The communication disturbances index. Unpublished administration manual. 1996.

[80] Schneider M, Reininghaus U, Van Nierop M, Janssens M, Myin-Germeys I. Does the social functioning scale reflect real-life social functioning? An experience sampling study in patients with a non-affective psychotic disorder and healthy control individuals. Psychol Med. 2017;47(16):2777–2786. 28534464 10.1017/S0033291717001295

[81] Cornblatt B, Auther A, Niendam T, Smith C, Zinberg J, Bearden C, et al. Preliminary findings for two new measures of social and role functioning in the prodromal phase of schizophrenia. Schizophr Bull. 2007;33(3):688–702. 17440198 10.1093/schbul/sbm029 PMC2526147

[82] Linscott R, van Os J. An updated and conservative systematic review and meta-analysis of epidemiological evidence on psychotic experiences in children and adults: on the pathway from proneness to persistence to dimensional expression across mental disorders. Psychol Med. 2013;43(6):1133–49. 22850401 10.1017/S0033291712001626

[83] Devoe D, Peterson A, Addington J. Negative symptom interventions in youth at risk of psychosis: a systematic review and network meta-analysis. Schizophr Bull. 2018;44(4):807–823. 29069511 10.1093/schbul/sbx139 PMC6007754

[84] Fusar-Poli P, Papanastasiou E, Stahl D, Rocchetti M, Carpenter W, Shergill S, et al. Treatments of negative symptoms in schizophrenia: meta-analysis of 168 randomized placebo-controlled trials. Schizophr Bull. 2015;41(4):892–899. 25528757 10.1093/schbul/sbu170 PMC4466178

[85] Harvey R, James A, Shields G. A systematic review and network meta-analysis to assess the relative efficacy of antipsychotics for the treatment of positive and negative symptoms in early-onset schizophrenia. CNS Drugs. 2016;30(1):27–39. 26801655 10.1007/s40263-015-0308-1

[86] Keefe R, Harvey P. Cognitive impairment in schizophrenia In: Geyer M, Gerhard G, editors. Novel antischizophrenia treatments, Berlin: Springer; 2012, p. 11–37. 10.1007/978-3-642-25758-2_2 23027411

